# Supplementary material for: Conservation of dynamic characteristics of transcriptional regulatory elements in periodic biological processes
Source: BMC Bioinformatics. 2022 Mar 17;23:94. doi: 10.1186/s12859-022-04627-9 (PMC8932128; doi:10.1186/s12859-022-04627-9)
Supplement: Supplementary file 5 — Additional file 5. Supplementary information: A PDF document with additional files including mathematical details, Figs. S1–S7 and Tables S1–S3. [file 12859_2022_4627_MOESM5_ESM.pdf]

Conservation of dynamic characteristics of transcriptional regulatory elements in periodic biological processes

Supplementary Information

Francis C. Motta[1*†], Robert C. Moseley[2†], Bree Cummins[3], Anastasia Deckard[4], and Steven B. Haase[2]

1 Department of Mathematical Sciences, Florida Atlantic University, Boca Raton, FL, USA
2 Biology, Duke University, Durham, NC, USA
3 Department of Mathematical Sciences, Montana State University, Bozeman, MT, USA
4 Geometric Data Analytics, Durham, NC, USA

[†] These authors contributed equally to this work.
*Correspondence: fmotta@fau.edu

Contents

Methods **2**
Dynamic Curve Features 2
De Lichtenberg Measures 2
JTK-CYCLE Measures 2
Precision-Recall Curves & Average Precision 3

Figure S1 – Precision-recall curves for *S. cerevisiae* microarray **4**

Figure S2 – Precision-recall curves for *S. cerevisiae* RNAseq **5**

Figure S3 – Precision-recall curves for *M. Musculus* microarray **6**

Figure S4 – Precision-recall curves for *M. Musculus* RNAseq **7**

Figure S5 – Precision-recall curves for *A. thaliana* microarray LDHC **8**

Figure S6 – Precision-recall curves for *A. thaliana* microarray LL_LDHC **9**

Figure S7 – Number of permutations vs. unique DL Reg p-values **10**

Table S1 – Ranks of core genes using the DL×JTK score **11**

Table S2 – Top 25 highest DL-ranked genes **12**

Table S3 – Top 25 highest PerReg-ranked genes **13**

References **14**

Methods

Dynamic Curve Features

De Lichtenberg Measures

In [1] the authors introduced a periodicity detection algorithm designed to identify genes in yeast that oscillated with the cell division cycle. The de Lichtenberg algorithm (DL) measures how periodic a signal is at a specified period by quantifying and combining statistical measures of gene expression periodicity and strength of regulation (Eq. 1). For each gene expression profile two empirical p-values, p_{reg} and p_{per}, are independently computed. Respectively, these p-values estimate the probabilities that the observed fold-change variability (Eq. 2) and rhythmicity at a specified period (Eq. 3) of the expression profile occurred, in some sense, at random. These statistics are then combined in a manner which accentuates expression profiles that are simultaneously highly-periodic and highly-variable. Explicitly, let $G \in \mathcal{G}$ be the gene expression profile, measured at timepoints $T = (t_1, \ldots, t_n)$, corresponding to gene G in the set of all measured gene expression profiles in a collection, $\mathcal{G}$. Then

$$\mathrm{DL}(G) := p_{\mathrm{reg}}(G) p_{\mathrm{per}}(G) \left[1 + \left(\frac{p_{\mathrm{reg}}(G)}{0.001}\right)^2\right] \left[1 + \left(\frac{p_{\mathrm{per}}(G)}{0.001}\right)^2\right]. \tag{1}$$

The p-value $p_{\mathrm{reg}}(G)$ is meant to be the probability that a "random" curve appears more highly regulated than G. This variability metric, denoted $\mathrm{Reg}(G)$, and defined by

$$\mathrm{Reg}(G) = \mathrm{std}\left(\log_{10}\left(G/\mathrm{mean}(G)\right)\right) \tag{2}$$

captures the deviation of the time series about its mean, with a small value indicating little variation over time about the mean expression level. Thus, $\mathrm{Reg}(G)$ may be interpreted as the magnitude of regulation of the gene G. The rationale of this study is rooted in the expectation that $\mathrm{Reg}(G)$ will actually be largest among those genes which are primarily responsible for generating an observed program of dynamic transcript abundance.

In practice, to estimate $p_{\mathrm{reg}}(G)$, an empirical null distribution of curve variability metrics is generated by first creating a large number of random expression curves through selection at each experimental time point the expression value of a transcript chosen from $\mathcal{G}$ uniformly at random. The variability metric (Reg) of each random curve is then computed and $p_{\mathrm{reg}}(G)$ is taken as the fraction of random curves whose regulation score is larger than $\mathrm{Reg}(G)$.

Likewise, for each gene G, many simulated curves are generated by randomly permuting the expression values of G and comparing the periodicity metric (called a Fourier score in [1]) of the randomized curve to that of G. The p-value $p_{\mathrm{per}}(G)$ is the fraction of simulated curves whose periodicity score (Per) is larger than that of G. This periodicity metric is taken to be

$$\mathrm{Per}(G) := \sqrt{(G \cdot \cos(\omega T))^2 + (G \cdot \sin(\omega T))^2}, \tag{3}$$

where ω is a specified period, and therefore reflects the magnitude of the Fourier coefficient at that period. A new implementation of the DL algorithm was written for use in this analysis and is available as a Python 3 module under the MIT License [2].

JTK-CYCLE Measures

JTK-CYCLE (JTK) was developed as a periodicity-detection algorithm to identify circadian rhythmically expressed genes in mice [3]. Since then it has been successfully

applied to time series transcriptomics data collected from many other species exhibiting rhythmic phenotypes. JTK correlates a gene's expression profile to that of a reference curve with known periodicity properties, and computes a significance of that correlation. Usually, and in this analysis, sinusoidal template curves are generated with user-specified periods and at various phase shifts determined by the sampling times of the expression profiles. Then the ordering of the time points by expression levels in a transcript abundance time series is compared to the orderings given by the template curves using Kendall's Tau coefficient [4]. Kendall's Tau measures the total number of agreements (concordances) and disagreements (discordances) in the relative orderings of each pair of time points given by the two ranking, and is therefore invariant to monotonic transformations of the data—making it well-suited to capture non-linear correlations. By precomputing the exact null distribution of Kendall's tau correlation using the Harding algorithm [5], an exact Bonferroni-adjusted p-value is rapidly computed for each gene, G, and is denoted here by $p_{jtk}(G)$. An implementation of JTK in Python by Alan Hutchinson [6] was modified for use in this analysis and is available as a Python 3 module under the MIT License [7].

Precision-Recall Curves & Average Precision

Given a ranking of N genes by some metric that is meant to discriminate between core and non-core, for some choice of threshold, we examine the true positives (TP) compared to false positives (FP) and/or false negatives (FN). The *precision* of the classifier's ranking at a given threshold is the fraction of true core genes among all genes ranked above that threshold, i.e. TP / (TP + FP). The *recall* is the fraction of true core genes appearing above the chosen threshold: TP / (TP + FN).

The *average precision* (AP) of the *precision-recall* (PR) curve is defined to be $\sum_{n=1}^{N} P_n \Delta R_n$, where ΔR_n is the change in the recall level caused by moving the decision threshold from the $(n-1)$st gene to the nth gene in the list of genes ranked by some metric, and P_n is the precision of the metric when the threshold is set to the metric of the nth gene in the ranked list.

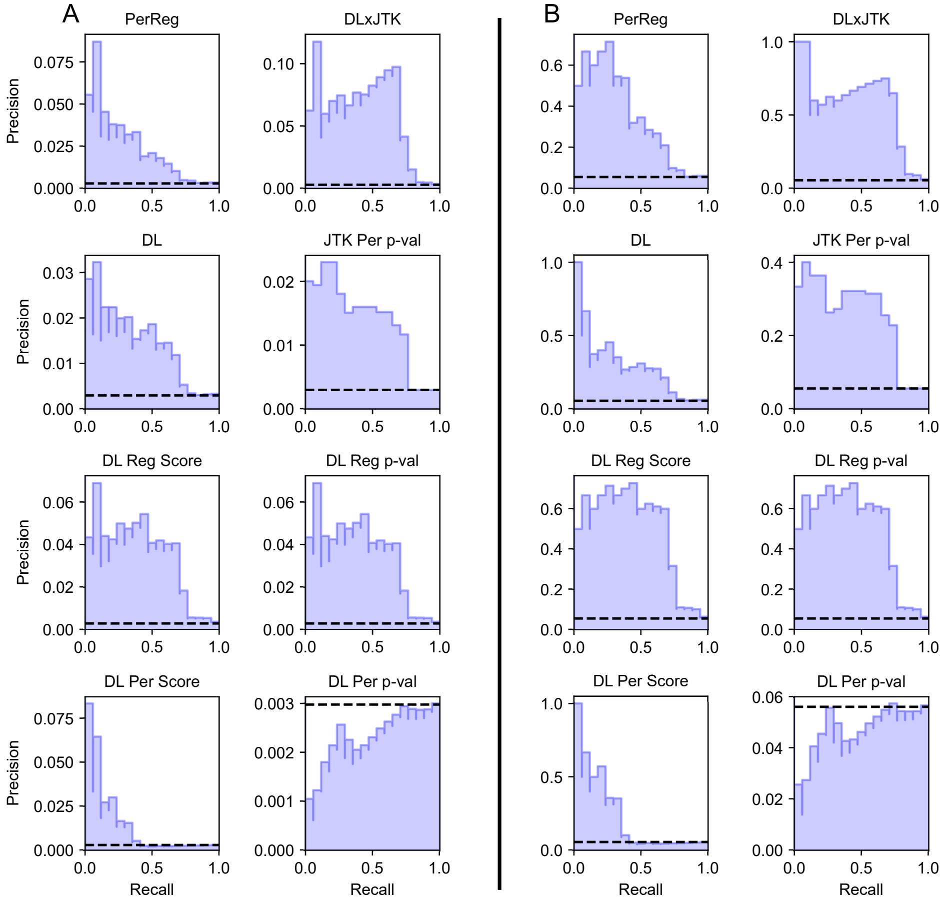

Figure S1. Precision-recall curves of classifiers identifying core from non-core TFs among all genes (A) and among only TFs (B) in *S. cerevisiae* microarray dataset. Between changes in recall, $R_{n-1} < R_n$, precision is plotted as a constant equal to the precision P_n at the minimum decision-threshold rank giving a recall of R_n, so that the area under the curve is equal to average precision. The horizontal dashed line indicates the baseline average precision of a random classifier.

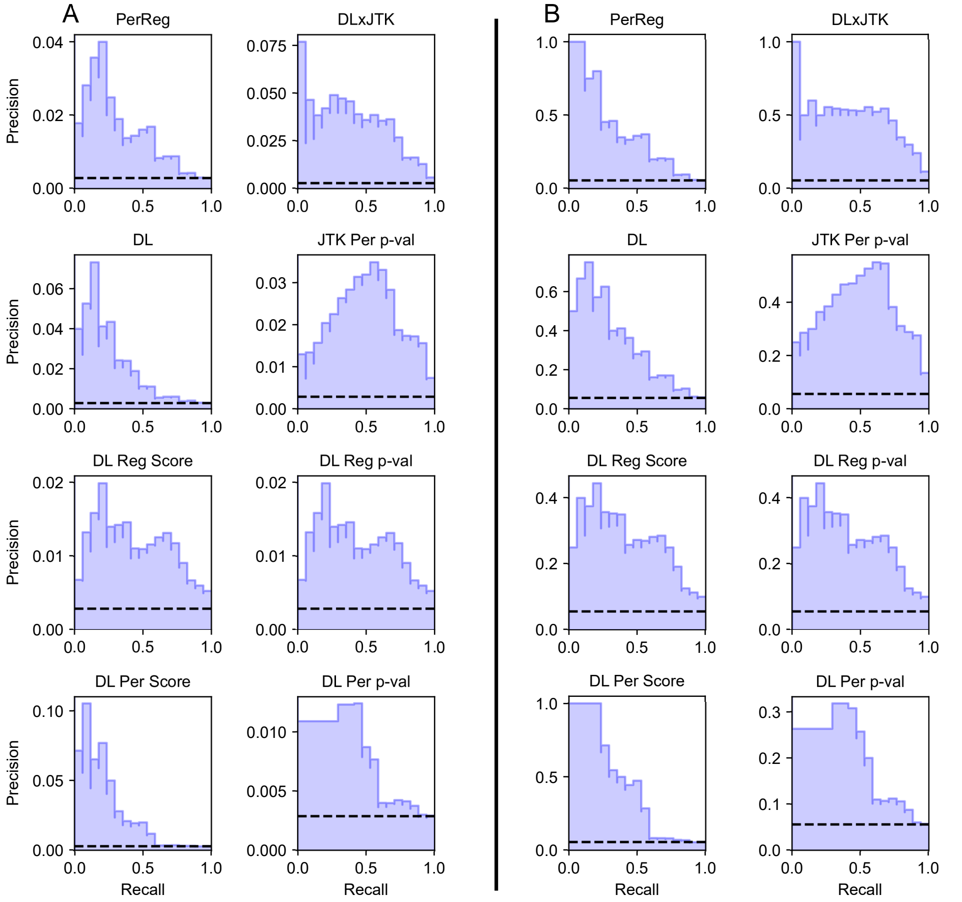

Figure S2. Precision-recall curves of classifiers identifying core from non-core TFs among all genes (A) and among only TFs (B) in *S. cerevisiae* RNAseq dataset. Between changes in recall, $R_{n-1} < R_n$, precision is plotted as a constant equal to the precision P_n at the minimum decision-threshold rank giving a recall of R_n, so that the area under the curve is equal to average precision. The horizontal dashed line indicates the baseline average precision of a random classifier.

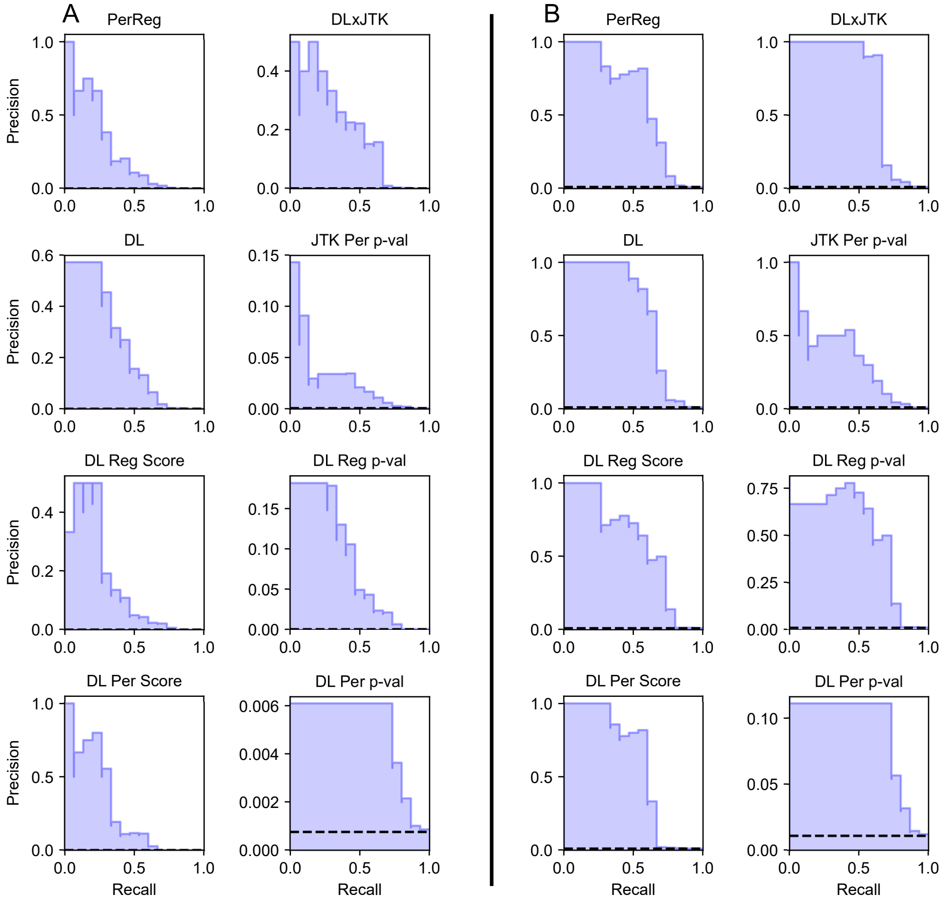

Figure S3. Precision-recall curves of classifiers identifying core from non-core TFs among all genes (A) and among only TFs (B) in *M. Musculus* microarray dataset. Between changes in recall, $R_{n-1} < R_n$, precision is plotted as a constant equal to the precision P_n at the minimum decision-threshold rank giving a recall of R_n, so that the area under the curve is equal to average precision. The horizontal dashed line indicates the baseline average precision of a random classifier.

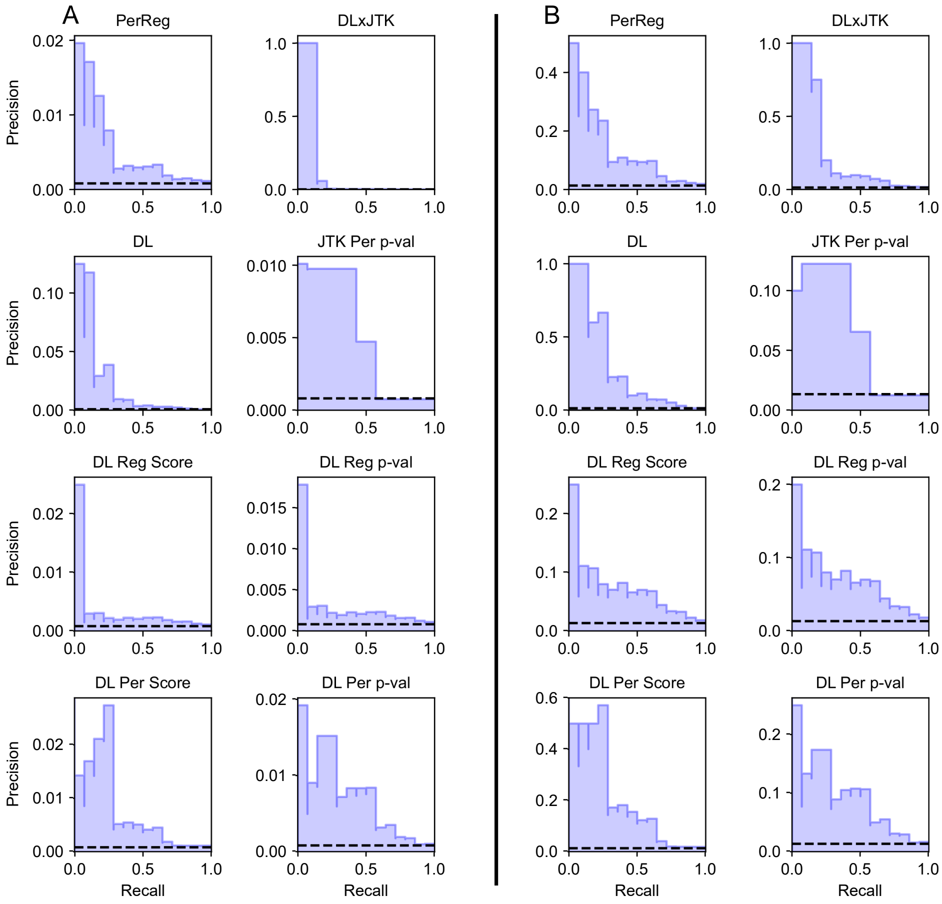

Figure S4. Precision-recall curves of classifiers identifying core from non-core TFs among all genes (A) and among only TFs (B) in *M. Musculus* RNAseq dataset. Between changes in recall, $R_{n-1} < R_n$, precision is plotted as a constant equal to the precision P_n at the minimum decision-threshold rank giving a recall of R_n, so that the area under the curve is equal to average precision. The horizontal dashed line indicates the baseline average precision of a random classifier.

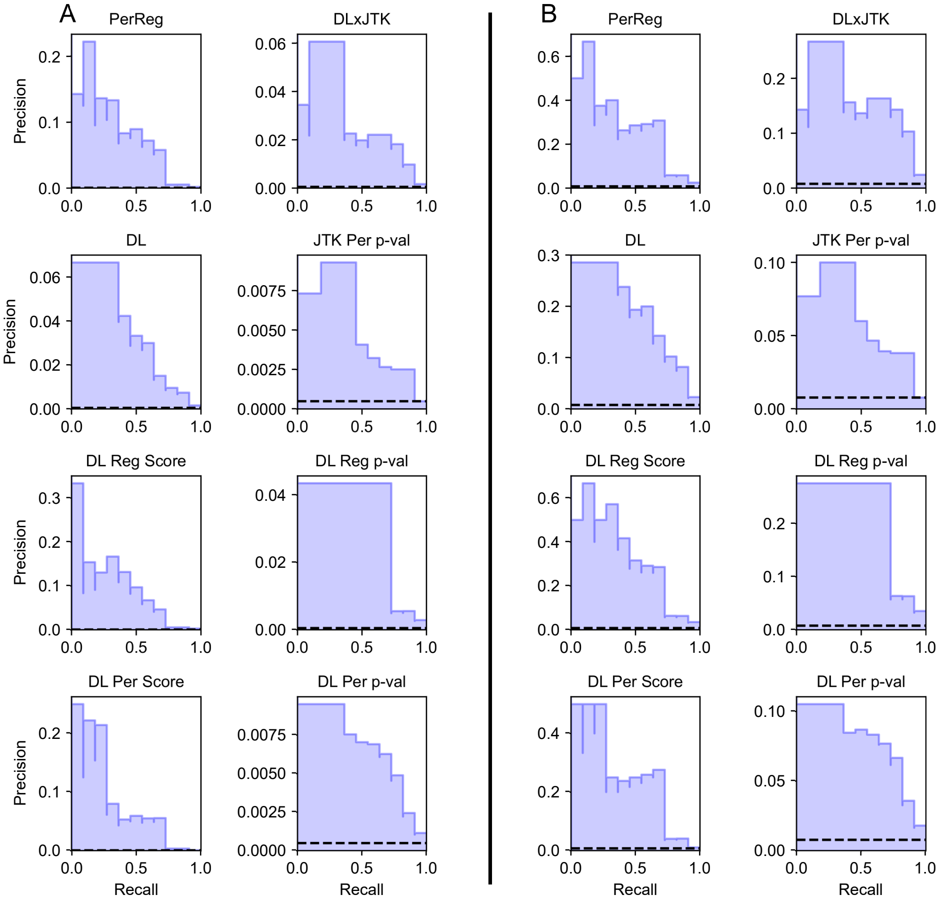

Figure S5. Precision-recall curves of classifiers identifying core from non-core TFs among all genes (A) and among only TFs (B) in *A. thaliana* microarray LDHC dataset. Between changes in recall, $R_{n-1} < R_n$, precision is plotted as a constant equal to the precision P_n at the minimum decision-threshold rank giving a recall of R_n, so that the area under the curve is equal to average precision. The horizontal dashed line indicates the baseline average precision of a random classifier.

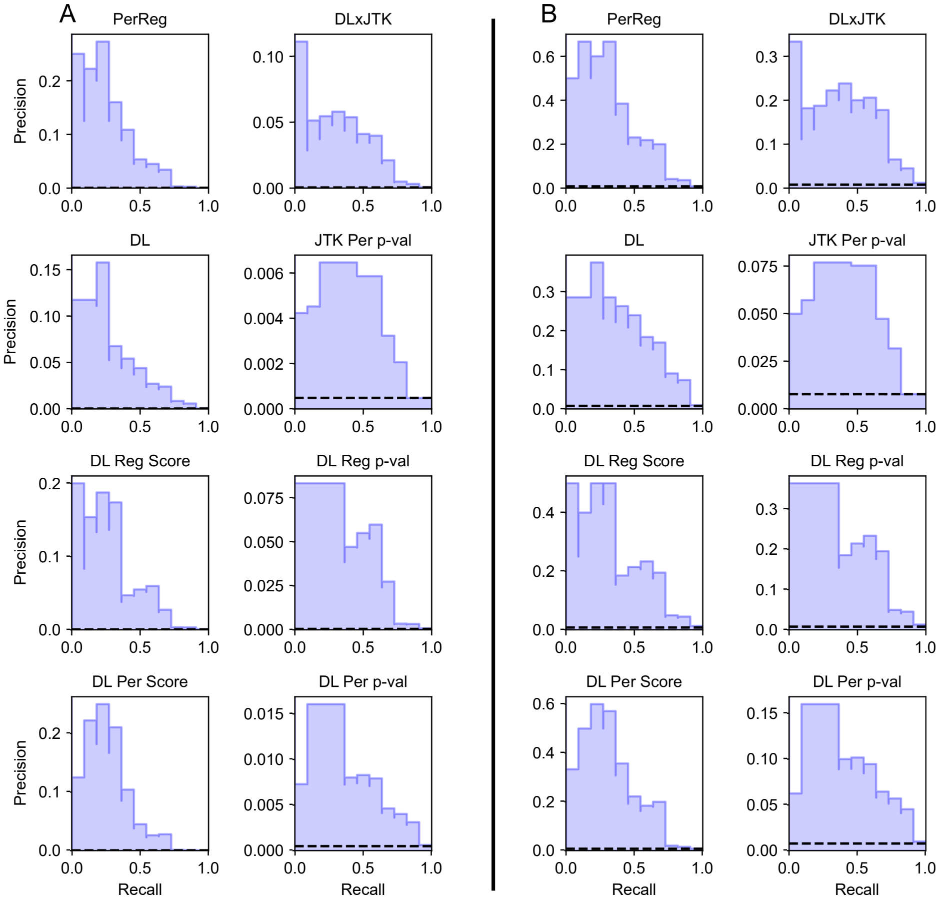

Figure S6. Precision-recall curves of classifiers identifying core from non-core TFs among all genes (A) and among only TFs (B) in *A. thaliana* microarray LL_LDHC dataset. Between changes in recall, $R_{n-1} < R_n$, precision is plotted as a constant equal to the precision P_n at the minimum decision-threshold rank giving a recall of R_n, so that the area under the curve is equal to average precision. The horizontal dashed line indicates the baseline average precision of a random classifier.

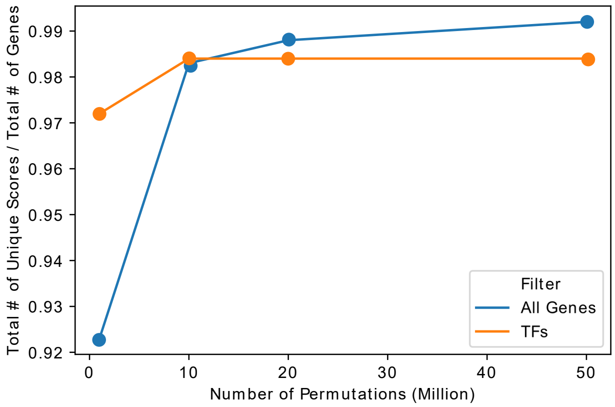

Figure S7. The number of permutations for computing DL Reg *p*-val vs. the number of unique DL Reg *p*-values. The number of unique values of DL Reg *p*-val per total number of genes before filtering for transcription factors (blue line) and after filtering for transcription factors (orange line) for the *A. thaliana* LDHC dataset as a function of the number of permutations chosen for the DL algorithm (1e6, 10e6, 20e6, or 50e6).

Table S1. Ranks among all transcription factors of core genes using the DL×JTK score.

S. cerevisiae			*M. Musculus*			*A. thaliana*		
Gene	MA	RNA	Gene	MA	RNA	Gene	LDHC	LL_LDHC
TOS4	16	1	*DBP*	2	1	*CCA1*	11	3
SWI5	1	4	*NPAS2*	3	2	*LHY*	6	11
YOX1	2	5	*ARNTL*	1	4	*LUX*	30	16
ASH1	14	8	*NR1D1*	4	20	*PRR7*	12	17
FKH1	10	9	*NR1D2*	5	45	*RVE8*	15	21
ACE2	5	11	*RORC*	10	67	*PRR9*	49	30
SWI4	12	13	*NFIL3*	8	71	*TOC1*	44	34
PLM2	11	15	*TEF*	11	87	*PRR5*	48	45
NDD1	13	17	*BHLHE41*	6	123	*CHE*	97	138
HCM1	8	18	*HLF*	69	164	*RVE4*	63	222
STB1	7	21	*CLOCK*	7	419	*RVE6*	453	908
YHP1	15	22	*BHLHE40*	195	501			
FKH2	49	28	*ARNTL2*	982	671			
FHL1	20	40	*RORA*	283	823			
SWI6	176	50	*RORB*	1210	NA			
MBP1	151	66						
MCM1	266	146						
**No. of TFs**†	304	307		1373	1118		1415	1415

LL_LDHC: Constant light and temperature; LDHC: 24 hour cycling light and temperature; MA: Microarray; RNA: RNAseq

† Counts are based on post-processed datasets (see Materials and Methods)

Table S2. The top 25 highest DL-ranked genes among all transcription factors.

Rank	*S. cerevisiae* MA	*S. cerevisiae* RNA	*M. Musculus* MA	*M. Musculus* RNA	*A. thaliana* LDHC	*A. thaliana* LL_LDHC
1	***ASH1****	*RME1*	***ARNTL****	***NPAS2****	***LHY****	*RVE1*
2	*RME1*	***YOX1****	***DBP****	***DBP****	*BBX19*	*PIF4*
3	***SWI5****	***ASH1****	***NPAS2****	*CDX4*	*COL2*	*BBX18*
4	*HST4*	***TOS4****	***NR1D1****	*GM14444*	***RVE8****	*COL5*
5	*NUT1*	*KAR4*	***BHLHE41****	***ARNTL****	***TOC1****	***LHY****
6	*HST3*	*RTT107*	***NR1D2****	***NR1D1****	*COL1*	*STH*
7	*CIN5*	***SWI5****	***NFIL3****	*CREB5*	*COL9*	***PRR7****
8	***ACE2****	***SWI4****	*EGR1*	*PPARD*	*STH*	***RVE8****
9	*MET4*	*TEC1*	***TEF****	*NPAS3*	*TGA3*	*COL1*
10	***SWI4****	*HST4*	*PPARD*	*POU4F1*	*RVE1*	*COL2*
11	***YOX1****	*ASF1*	***RORC****	*FOXO6*	*HYH*	*AT2G28200*
12	*ISW2*	*HST3*	*MAFB*	*DMRTA2*	*BBX18*	*PIL6*
13	*RGT2*	*RLF2*	*KLF10*	*EGR1*	*AT1G26790*	*MYBL2*
14	*MET18*	*WTM1*	*RFXANK*	*GM20422*	***PRR7****	***CCA1****
15	*RGT1*	***ACE2****	***CLOCK****	*EGR3*	*HB-12*	*BBX8*
16	*SIP4*	*ZNF1*	*TSC22D3*	*TBX1*	*PRE1*	*HSFA8*
17	***TOS4****	***STB1****	*KLF13*	*MAFF*	*STO*	*CDF3*
18	*RTT107*	*WTM2*	*NR0B2*	*GM6710*	*PIL6*	*ABF1*
19	*ESC2*	*CSE2*	*ZFP36l1*	*EN2*	***CCA1****	***TOC1****
20	*ASF1*	*GAT1*	*ATF5*	*ZBTB7C*	*EPR1*	*EPR1*
21	*WTM2*	*OTU1*	*SREBF1*	*MESP1*	*LZF1*	*AT1G70000*
22	*HAP5*	***HCM1****	*STAT5B*	***NR1D2****	*BZS1*	*STO*
23	*HPA2*	*PHD1*	*GTF2IRD1*	*ZFP987*	*ASG4*	*BBX16*
24	*EDS1*	*MAL13*	*ZBTB21*	*GM14401*	*CDF3*	*ATCTH*
25	*RTG2*	*RGT2*	*SOX9*	*GM14305*	*CO*	***LUX****
Recall	35.3%	47.1%	66.7%	35.7%	45.5%	54.5%

LL_LDHC: Constant light and temperature; LDHC: 24 hour cycling light and temperature; MA: Microarray; RNA: RNAseq

***** Core transcription factors in Dataset S3.

Table S3. The top 25 highest PerReg-ranked genes among all transcription factors.

Rank	*S. cerevisiae*		*M. Musculus*		*A. thaliana*	
	MA	RNA	MA	RNA	LDHC	LL_LDHC
1	*RME1*	***YOX1****	***NR1D1****	*CDX*	*RVE1*	*MYBL2*
2	***ASH1****	***TOS4****	***NPAS2****	***NPAS2****	***LHY****	***CCA1****
3	***SWI5****	*RME1*	***DBP****	*GM14444*	***CCA1****	***LHY****
4	*HST3*	***ASH1****	***ARNTL****	*FOXO6*	*COL2*	*COL2*
5	***ACE2****	***SWI5****	*EGR1*	***NR1D1****	*STH*	***PRR7****
6	***SWI4****	*KAR4*	***NR1D2****	*POU4F1*	*MYBL2*	***PRR5****
7	***YOX1****	*HST3*	*MYC*	*GM20422*	*AT1G26790*	*PIF4*
8	*HST4*	*ZNF1*	***NFIL3****	*DMRTA2*	***PRR7****	*STH*
9	*SIP4*	*RTT107*	***BHLHE41****	*NPAS3*	*HYH*	*COL5*
10	*ASF1*	*MGA1*	***TEF****	*EGR1*	***PRR5****	*RVE1*
11	***TOS4****	***SWI4****	***RORC****	***DBP****	*BBX16*	*CDF3*
12	*RTT107*	*SIP4*	*ZBTB16*	*CREB5*	*LZF1*	*LZF1*
13	***STB1****	***ACE2****	*PPARD*	*TBX1*	*BBX18*	***LUX****
14	*EDS1*	*PHD1*	*MAFB*	*EGR3*	*CDF2*	*CDF1*
15	*GAT1*	*MAL33*	*KLF10*	*MESP1*	*EPR1*	*COL1*
16	*HAP4*	*HST4*	*NR0B2*	*GM6710*	*CDF1*	*ATCTH*
17	*HPA2*	*TEC1*	*RFXANK*	***ARNTL****	*COL1*	*CDF2*
18	*GAL3*	*RLF2*	*BCl6*	*EN2*	*BBX19*	*BBX18*
19	*WTM1*	*ASF1*	*ZFP931*	*ZBTB7C*	***LUX****	*ABF1*
20	*RLF2*	***FKH1****	*NR3C2*	*GM14401*	*PIF4*	*COL9*
21	*NDT80*	*MSS11*	***CLOCK****	*GM14305*	***PRR9****	*EPR1*
22	*XBP1*	*MTH1*	*ONECUT1*	*BHLHE22*	*CDF3*	*AT1G26790*
23	*TEC1*	*RPI1*	*KLF9*	*PPARD*	*RVE2*	*BBX16*
24	*MSN4*	***STB1****	*ZBTB7C*	*OVOl2*	***RVE8****	*AT5G44260*
25	***HCM1****	***HCM1****	*FOXQ1*	*FOXP3*	*TGA3*	*HYH*
Recall	47.1%	52.9%	66.7%	28.6%	63.6%	45.5%

LL_LDHC: Constant light and temperature; LDHC: 24 hour cycling light and temperature; MA: Microarray; RNA: RNAseq
***** Core transcription factors in Dataset S3.

References

1. de Lichtenberg U, Jensen LJ, Fausbøll A, Jensen TS, Bork P, Brunak S. Comparison of computational methods for the identification of cell cycle-regulated genes. Bioinformatics. 2005;21(7):1164–1171. doi:10.1093/bioinformatics/bti093.

2. Motta F, Deckard A, Moseley RC. pyDL; 2019. `https://gitlab.com/biochron/pydl`.

3. Hughes ME, Hogenesch JB, Kornacker K. JTK_CYCLE: An Efficient Nonparametric Algorithm for Detecting Rhythmic Components in Genome-Scale Data Sets. Journal of Biological Rhythms. 2010;25(5):372–380. doi:10.1177/0748730410379711.

4. Kendall MG. A NEW MEASURE OF RANK CORRELATION. Biometrika. 1938;30(1-2):81–93. doi:10.1093/biomet/30.1-2.81.

5. Harding EF. An Efficient, Minimal-Storage Procedure for Calculating the Mann-Whitney U, Generalized U and Similar Distributions. Journal of the Royal Statistical Society Series C (Applied Statistics). 1984;33(1):1–6.

6. Hutchison A. pyJTK: Python implementation of the JTK_CYCLE statistical test; 2013. `https://github.com/alanlhutchison/pyJTK`.

7. Hutchinson A, Motta F, Deckard A. pyJTK; 2019. `https://gitlab.com/biochron/pyjtk`.
